# N-myc downstream-regulated gene 2 (*NDRG2*) promoter methylation and expression in pituitary adenoma

**DOI:** 10.1186/s13000-017-0622-7

**Published:** 2017-04-08

**Authors:** Paulina Vaitkiene, Indre Valiulyte, Brigita Glebauskiene, Rasa Liutkeviciene

**Affiliations:** Neuroscience Institute, Lithuanian University of Health Sciences, Eiveniu str.4, LT-50009 Kaunas, Lithuania

**Keywords:** Pituitary adenoma, N-myc downstream-regulated gene 2, *NDRG2*, DNA methylation, mRNA expression, Invasiveness

## Abstract

**Background:**

Pituitary adenoma (PA) is a benign primary tumor that arises from the pituitary gland and is associated with ophthalmological, neurological and endocrinological abnormalities. However, causes that increase tumor progressing recurrence and invasiveness are still undetermined. Several studies have shown N-myc downstream regulated gene 2 (*NDRG2*) as a tumor suppressor gene, but the role of *NDRG2* gene in pituitary adenoma pathogenesis has not been elucidated. The aim of our research has been to examine *NDRG2* mRNA expression in PA and to determine the associations between the *NDRG2* gene epigenetic changes and the development of recurrence or invasiveness of PA and patient clinical data.

**Methods:**

The MS-PCR was used for *NDRG2* promoter methylation analysis and gene mRNA expression levels were evaluated by qRT-PCR in 68 non-functioning and 73 functioning adenomas. Invasiveness was evaluated using magnetic resonance imaging with Hardy’s modified criteria. Statistical analysis was performed to find correlations between *NDRG2* gene mRNA expression, promoter methylation and patient clinical characteristics and PA activity.

**Results:**

The *NDRG2* mRNA expression was significantly lower in the case of acromegaly (GH and IGF-1 hypersecretion) than in other diagnoses of PAs (*p* < 0.05). Also, the *NDRG2* expression was significantly higher in prolactinoma (PRL hypersecretion) than in in other diagnoses of PAs (*p* < 0.05). The promoter of *NDRG2* was methylated in 22.69% (12/58 functioning and 15/61 non-functioning) of patients with PA. However, the *NDRG2* gene mRNA expression was not significantly related to its methylation status. Clinical factors, such as: age, gender, relapse and diagnoses of Cushing syndrome were of no significance for *NDRG2* promoter methylation and mRNA expression levels, as well as secreting or non-secreting PAs and the invasiveness of PAs.

**Conclusion:**

The different NDRG2 promoter methylation and expression levels in PA samples showed tumor heterogeneity and indicates a potential role of this gene in pituitary adenoma pathogenesis, but the corresponding details require intensive research.

## Background

Pituitary adenoma (PA) is a common intracranial neoplasm with reported estimated prevalence rates to be 14.4% to 22.5% in pooled autopsy and radiological series, respectively [1]. PAs are generally benign but can behave clinically in different ways. Some of pituitary tumors are hormonally inactive, others secrete hormones in excess, and some of PAs can cause morbidity because of dysregulation of hormone production and/or symptoms of mass effect [2, 3]. The pituitary gland is localized in a dual bag attached to the inferior aspect of the diaphragm of the sella and surrounded by venous spaces that correspond laterally to the cavernous sinuses [4]. Cavernous sinus invasion has an influence on the management and prognosis of PA [5], because dual wall invasion usually implies partial surgical removal of the tumor [6]. Early prediction of which pituitary tumor will recur and/or exhibit an invasive phenotype remains difficult despite the introduction of several tissue-based molecular markers [7].

Associations between tumors (including glioblastoma, gastric cancer, colorectal cancer, breast and liver cancers, meningioma, bladder and thyroid cancers) and N-myc downstream-regulated gene 2 (*NDRG2*) have been reported in numerous studies [8–16]. *NDRG2* is a member of the NDRG family, which consists of *NDRG1, NDRG2, NDRG3* and *NDRG4* [17] and is located at chromosome 14q11.2, a region that has been reported to harbor a tumor suppressor gene [18]. *NDRG2* is highly expressed in the brain and skeletal muscle, while it is marginally expressed or almost undetectable in the several human cancer cell lines [17, 19]. NDRG proteins are reported to be involved in cell proliferation, differentiation, migration, invasion and stress response [19]. It was shown that NDRG2 reduce tumor cell proliferation in glioblastomas [20]. Also, NDRG2 upregulation was associated with Alzheimer's disease or cerebral ischemia [21, 22]. Several studies have shown *NDRG2* promoter CpG island methylation and down-regulation in liver [13], gastric [10], colorectal cancers (CRC) [23, 24], glioblastomas [8, 9] and anaplastic meningioma [25]. However, *NDRG2* promoter methylation and mRNA expression levels in PAs has not been investigated.

The aim of this study was to determinate aberrant promoter methylation and mRNA expression of *NDRG2* in PAs and to evaluate the associations between the methylation profile of gene, mRNA expression, patients’ clinical characteristics and tumor invasiveness and recurrence.

## Methods

### Description of subject

One hundred forty one pituitary adenoma tissues and clinical patient data were collected at the Department of Neurosurgery of Hospital of Lithuanian University of Health Sciences between 2010 and 2016. Tumor tissues were frozen in liquid nitrogen immediately after their surgical resection. The age at the time of the operation, gender, relapse, size and diagnoses of Cushing syndrome, acromegaly or prolactinoma were collected for each patient. The endocrinological features were: 73 functioning and 68 nonfunctioning adenomas. According to the clinical findings functioning adenomas were: 7 growth hormone (GH) - secreting adenomas, 2 insulin-like grow factor 1 (IGF-1) - secreting adenomas, 1 cortisol (COR) - secreting adenoma, 44 prolactin (PRL) - secreting adenomas, 1 adrenocorticotropic hormone (ACTH) - secreting adenoma and 18 adenomas secreting more than one hormone. According to tumor size all PAs were macroadenomas (greater than 10 mm).

Invasion of pituitary adenomas were analyzed using MR imaging findings and classified according to Hardy classification, modified by Wilson [5]. The Knosp classification system was used to quantify the invasion of the cavernous sinus [6]. Invasiveness was established in 71 patients with pituitary adenoma. From them, 51 invasive and 20 non-invasive PAs were found.

### Nucleic acid extraction

Tissue specimens were pulverized and stored at -80 °C until DNA and RNA was obtained. Genomic DNA was extracted from 119 PA specimens by SDS/proteinase K treatment, followed by phenol–chloroform extraction and ethanol precipitation. The remaining 22 samples were missing because of containing too small an amount of tumor tissue. All the samples were stored at -20 °C until DNA was modified with sodium bisulfite.

Total RNA was extracted from 141 PAs using Trizol reagent, according to the manufacturer’s protocol (Ambion, Life Technologies) and stored at -80 °C until cDNA synthesis. However, 10 mRNA samples were lost because the concentrations for cDNA synthesis were too small. The genomic DNA and RNA concentrations and purity was determined using Nanodrop spectrophotometer (Eppendorf). For pure DNA, A260/280 is ~1.8 and for pure RNA A260/280 is ~2.

### Bisulfite Modification and MS-PCR

Extracted genomic DNA of 119 PA samples was modified with EZ DNA methylation kit™ (Zymo Research), according to the manufacturer’s instructions. The sodium bisulfite treated DNA was eluted in 40 μL of nuclease-free water.

After bisulfite modification, the methylation-specific polymerase chain reactions (MS-PCR) were performed in 15 μl of 7.5 μL Maxima® Hot Start PCR Master Mix (ThermoFisher Scientific) with Hot Start Taq DNA polymerase, 10 pmol of each primer (Metabion International AG) and nuclease-free water. Primers for methylated *NDRG2* allele were: 5'-AGAGGTATTAGGATTTTGGGTACG-3' (forward) and 5'-GCTAAAAAAACGAAAATCTCGC-3' (reverse) and for unmethylated allele: 5'-AGAGGTATTAGGATTTT GGGTATGA-3' (forward) and 5'-CCACTAAAAAAACAAAAATCTCACC-3' (reverse), according to the published data [23]. The reaction was hotstart at 95 °C for 5 min. The amplifications were carried out in a thermal cycler (Eppendorf) for 38 cycles, each of which consisted of denaturation at 92 °C for 15 s, annealing at 60 °C for 30 s, and extension at 72 °C for 15 s, followed by a final 5 min extension at 72 °C. For each set of methylation-specific PCR reactions methylated (Bisulfite-Converted Universal Methylated Human DNA Standard (Zymo Research, USA)), unmethylated (human blood lymphocyte DNA, treated with bisulfite) and negative (nuclease-free water) controls were included in all reactions.

The MS-PCR products were analyzed by electrophoresis on a 2% agarose gel stained with ethidium bromide and visualized under UV illumination.

### cDNA synthesis and qRT-PCR

First-strand cDNA was produced from total RNA by using RevertAid H Minus M-MuLV Reverse Transcriptase (ThermoFisher Scientific) and random hexamer primers (ThermoFisher Scientific), according to the manufacturer’s protocol. Negative controls were prepared as above, but without Reverse Transcriptase.

For the *NDRG2* gene mRNA expression, Quantitative real-time PCR (qRT-PCR) was performed using the SYBR Green chemistry in a Real-Time PCR System “Applied Biosystems 7500 Fast” (Applied Biosystems, USA). The 12 μl reaction mixture contained of 6 μl Maxima SYBR Green/ROX qPCR Master Mix (2×) (ThermoFisher Scientific), 15 ng of the cDNA, nuclease-free water and gene-specific primers: *NDRG2* forward 5`-AGAGCTACGACCTGAC-3`, reverse 5`-AGCACTGTGTGTACAG-3` resulting in a 128 bp PCR amplicon to a total concentration of 0.6 μM. The housekeeping gene *β-actin* was used as an internal control with primers: forward 5`-CATTACACATCCAACC-3`, reverse 5`-GGAGTCAGCCTGAGGA-3`, resulting in a 184 bp PCR amplicon to a total concentration of 0.1 μM. The *NDRG2* and *β-actin* primers were designed according to the published data [26]. The PCR amplification was performed after denaturation step at 95 °C for 10 min followed by 40 cycles, each of which consisted of denaturation at 92 °C for 30 s, annealing at 60 °C for 30 s, and extension at 72 °C for 30 s, and a final step for the generation of a melting curve to distinguish between the main PCR product and primer-dimers. All measurements were performed in triplicate.

The comparative 2^-ΔΔCt^ method was used for the calculations of *NDRG2* gene mRNA expression. The comparison was carried out between PA normalized threshold cycle (Ct) values and healthy human brain (RHB) normalized Ct values: ΔΔCt = (Ct,_*NDRG2*_ - Ct,_*β-actin*_)_PA sample x_ - (Ct,_*NDRG2*_ - Ct,_*β-actin*_)_RHB_ [27]. The final result was given as log2(2^-ΔΔCt^) calculation.

For standard curve design, RHB “FirstChoice Human Brain Reference RNA” (Ambion, cat. No. AM6050) was used. Standard curve parameters for *NDRG2* were: efficiency 99.71%, R^2^ 0.994, slope −3.33; for *β-actin* were: efficiency 100.08%, R^2^ 0.997, slope −3.32.

### Statistical analysis

The SPSS Statistics 19 (SPSS Inc., Chicago, IL) software package was used for statistical analysis. Chi-square test was used to evaluate associations among *NDRG2* gene promoter methylation, mRNA expression levels and clinical characteristics (age, gender, relapse, Cushing syndrome, acromegaly, prolactinoma, invasiveness, secreting and non-secreting pituitary adenomas and hormone groups). The correlation between *NDRG2* gene expression and methylation and the other clinical factors were evaluated by use of the Mann-Whitney test. Kruskal–Wallis test was used to reveal the difference across medians of *NDRG2* mRNA expression in all hormone groups. The significance level was defined as *p* value less than 0.05.

## Results

### *NDRG2* gene methylation frequency in PAs and associations with patient clinical data

**Fig. 1:**
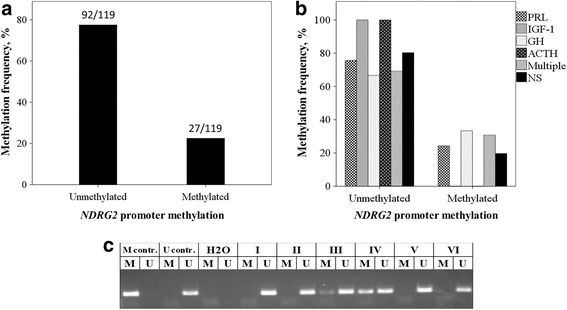
Analysis of *NDRG2* gene promoter methylation in PA samples. **a** Methylation frequency (%). **b** Hormone distribution of PAs in methylated and unmethylated *NDRG2* promoter groups. PRL – prolactin, IGF-1 – insulin-like grow factor 1, GH – growth hormone, ACTH – adrenocorticotropic hormone, multiple – PAs secreting more than one hormone, NS – non-secreting PAs. **c** Representative MS-PCR for *NDRG2* in PA samples. M indicates amplification of methylated alleles, U unmethylated alleles. M cont. – positive methylation control (Standard Bisulfite Converted Universal Methylated Human DNA), U cont. – negative methylation control (normal human peripheral lymphocytes), H2O – water control, I-VI designate PA samples

Methylation specific PCR analysis was performed to determine the methylation status of *NDRG2* gene in 119 PA samples (Fig. 1c). The gene was methylated in 22.69% (27/119) of cases. Representative chart is shown in Fig. 1a. These results indicate that *NDRG2* gene has low methylation status in PAs.

To characterize the correlation between methylation of *NDRG2* promoter and clinical features of PAs, several clinicopathological characteristics including age, gender, relapse, invasiveness, diagnoses of acromegaly, prolactinoma and Cushing syndrome were compared between methylated and unmethylated *NDRG2* promoter groups. However, Chi-square test showed that methylation of *NDRG2* promoter was not associated with any of this clinical data (*p* > 0.05) (Table 1).

**Table 1:** Relationship between *NDRG2* promoter methylation, patient clinical characteristics, PA invasiveness and *NDRG2* mRNA expression

	*NDRG2* gene methylation			
	Number of patients	M (%)	U (%)	*p*-value
Cases	119	27 (22.69)	92 (77.31)	
Age (years)				
≤60	48	13 (27.08)	35 (72.92)	0.368
>60	70	14 (20.00)	56 (80.00)	
Gender				
Female	68	15 (22.06)	53 (77.94)	0.850
Male	51	12 (23.53)	39 (76.47)	
PA function				
Secreting	61	15 (24.59)	46 (75.41)	0.612
Non-secreting	58	12 (20.69)	46 (79.31)	
Relapse				
Appear	10	2 (20.00)	8 (80.00)	0.832
None	109	25 (22.94)	84 (77.06)	
Prolactinoma				
Appear	37	9 (24.32)	28 (75.68)	0.952
None	24	6 (25.00)	18 (75.00)	
Acromegaly				
Appear	12	4 (33.33)	8 (66.67)	0.433
None	49	11 (22.45)	38 (77.55)	
Cushing syndrome				
Appear	1	0 (0.00)	1 (100.00)	0.565
None	60	15 (25.00)	45 (75.00)	
Hormones				
PRL	37	9 (24.32)	28 (75.68)	0.801
IGF-1	2	0 (0.00)	2 (100.00)	0.401
GH	6	2 (33.33)	4 (66.67)	0.639
ACTH	1	0 (0.00)	1 (100.00)	0.556
Multiple	13	4 (30.77)	9 (69.23)	0.616
Invasiveness				
Invasive	41	10 (24.39)	31 (75.61)	0.452
Non-invasive	19	3 (15.79)	16 (84.21)	
*NDRG2* mRNA expression				
Low	22	3 (13.64)	19 (86.36)	
Medium	50	13 (26.00)	37 (74.00)	0.458
High	37	7 (18.92)	30 (81.08)	

*M* methylated, *U* unmethylated, *PRL* prolactin, *IGF-1* insulin-like grow factor 1, *GH* growth hormone, *ACTH* adrenocorticotropic hormone, *multiple* PAs secreting more than one hormone

We then analyzed the relationship between *NDRG2* promoter methylation and PA hormonal activity (Fig. 1b). The histogram of hormones distribution in methylated and unmethylated gene groups revealed that hypersecretion of PRL, IGF-1, GH, ACTH and more than one hormone mostly occur in PAs with unmethylated promoter of *NDRG2* gene. Also, in most cases nonfunctioning PAs have unmethylated *NDRG2* gene. However, the data showed no statistically significant differences between these groups (*p* > 0.05) (Table 1).

### *NDRG2* gene expression in PAs and associations with *NDRG2* promoter methylation and patient clinical data

The expression of *NDRG2* mRNA in 131 PAs was determined by qRT-PCR method using the SYBR Green chemistry. The values were normalized with internal *β-actin* control. To investigate the correlation between *NDRG2* promoter methylation and mRNA expression, statistical analysis was performed in 109 PA samples that have the values of methylation and expression. However, Mann-Whitney test showed no significant differences of *NDRG2* mRNA expression between the group of *NDRG2* methylation samples (23 tumors) and the group of unmethylated *NDRG2* gene (86 tumors, *p* = 0.323).

**Fig. 2:**
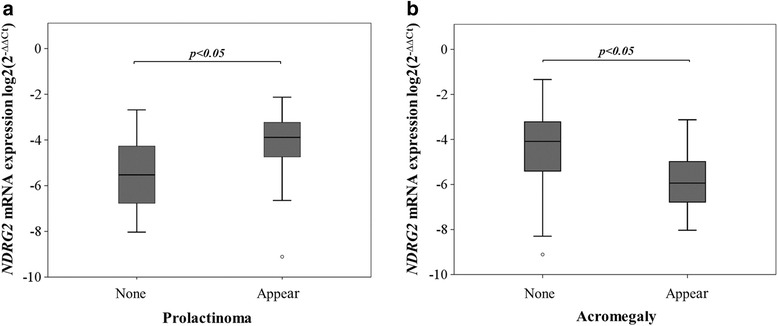
*NDRG2* gene expression associations with diagnoses of prolactinoma and acromegaly. **a** Box plots of relative *NDRG2* expression associated with diagnoses of prolactinoma. **b** Box plots of relative *NDRG2* expression associated with diagnoses of acromegaly. None – no diagnoses of acromegaly. The line inside each box represents the median, the lower and upper edges of the boxes represent the 25th and 75th percentiles, respectively, and upper and lower lines outside the boxes represent minimum and maximum values (error bars). The Mann-Whitney test showed, that the *NDRG2* expression level was increased with diagnoses of prolactinoma and decreased with diagnoses of acromegaly (*p* < 0.05)

We then analyzed the correlation between *NDRG2* gene mRNA expression and patient clinical characteristics. Using the Mann-Whitney test, we found that the expression of *NDRG2* had no correlation with age, gender, presence or absence of repeated surgery, secreting or non-secreting PAs and Cushing syndrome (*p* = 0.076, *p* = 0.545, *p* = 0.783, *p* = 0.927 and *p* = 0.980, respectively). Nevertheless, *NDRG2* expression was increased with diagnoses of prolactinoma and decreased with diagnoses of acromegaly, compared to patients with other symptoms (*p* < 0.05) (Fig. 2a, b).

**Fig. 3:**
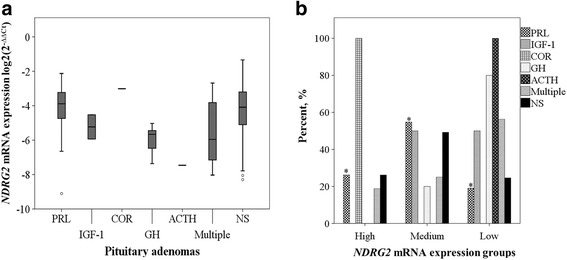
*NDRG2* gene expression associations with PAs hormones. **a** Box plots of medians of *NDRG2* mRNA expression in PRL, IGF-1, GH, ACTH, COR, multiple hormone groups and nonfunctioning PAs. The line inside each box represents the median, the lower and upper edges of the boxes represent the 25th and 75th percentiles, respectively, and upper and lower lines outside the boxes represent minimum and maximum values (error bars). PRL – prolactin, IGF-1 – insulin-like grow factor 1, GH – growth hormone, ACTH – adrenocorticotropic hormone, multiple – PAs secreting more than one hormone, NS – non-secreting PAs. **b** Hormones distribution in low, medium and high *NDRG2* expression levels. Significantly increased amount of PRL hormone was observed at medium *NDRG2* mRNA expression level (* *p* < 0.05)

To further investigate whether *NDRG2* gene mRNA expression was associated with hormones, we compared medians of *NDRG2* mRNA expression in PRL, IGF-1, GH, ACTH, COR and multi hormone groups and nonfunctioning PAs (Fig. 3a). Kruskal–Wallis test revealed that the medians of *NDRG2* mRNA expression in all hormone groups statistically differ (*p* < 0.05), and the box plots showed the tendency that GH-secreting, ACTH-secreting and more than one hormone secreting PAs are at lower gene expression level than other hormone groups. To specify these findings, we then analyzed whether the hormone groups and nonfunctioning PAs reflect on expression levels of *NDRG2* gene. For this matter, the *NDRG2* mRNA expression values were divided into three gene mRNA expression groups: “low” - values 1.0-fold lower than the *NDRG2* gene mRNA expression median, “medium” - values ranging in between “low” and “high”, and “high” - values 1.0-fold higher than the gene median) As shown in Fig. 3b, distribution of hormones in *NDRG2* expression levels were different. In most cases PRL hypersecretion was detected with medium *NDRG2* gene expression level (*p* < 0.05). IGF-1, COR, GH, and ACTH hormones showed no statistically significant differences (*p* = 0.730, *p* = 0.172, *p* = 0.075, *p* = 0.379, respectively) as well as multiple (more than one hormone) and non-secreting PAs (*p* = 0.096, *p* = 0.584, respectively). Nevertheless, the histogram of hormone groups distribution showed a tendency, that the hypersecretion of IGF-1 hormone mostly occur with medium and low *NDRG2* gene expression levels, COR - with high *NDRG2* mRNA expression level, GH, ACTH and multiple – at low gene expression level (Fig. 3b).

**Fig. 4:**
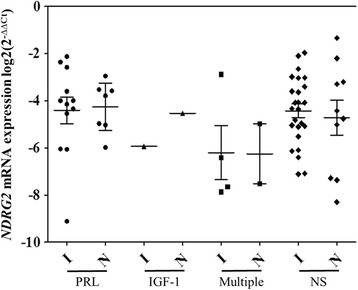
Dot plot analysis of *NDRG2* mRNA expression in invasive and noninvasive hormones of PAs. The horizontal bars represent the mean values with 95% confidence interval, the spots represent the amount of samples. PRL – prolactin, IGF-1 – insulin-like grow factor 1, multiple – PAs secreting more than one hormone, NS – non-secreting PAs; I – invasive, N – non-invasive PA

Finally, we analyzed the relationship between *NDRG2* gene promoter methylation, mRNA expression, and invasiveness of 71 pituitary adenomas (51 invasive and 20 noninvasive). However, the analysis (Chi-square and Mann-Whitney test) showed no significant differences between these groups (*p* = 0.452, *p* = 0.472, respectively). Additionally, secreting and non-secreting PAs also had no correlations with invasiveness (*p* = 0.571). We also wanted to reveal the tendency of invasiveness in PRL, IGF-1, GH, COR, ACTH and more than one hormone secreting PAs. In addition to this, the dot plot analysis was performed (Fig. 4). The comparison showed that the means of *NDRG2* gene mRNA expression of hormones in invasive and non-invasive tumor groups were various. The lowest *NDRG2* expression was detected in PAs secreting more than one hormone, and the highest in PRL-secreting and non-secreting pituitary adenomas (Fig. 4).

## Discussion

Pituitary adenoma is a common benign monoclonal neoplasm [28]. Early prediction of which pituitary tumors will recur and/or exhibit an invasive phenotype remains difficult [7]. *NDRG2* gene may be a promising target for cancer, because *NDRG2* down-regulation is associated with cancer development and progression, including such features as malignant clinical manifestations and increased pathological grade. Moreover, this gene is a relevant biomarker for predicting aggressive behavior, tumor recurrence and overall patient survival [29]. Therefore, should be further studies to show that NDRG2 up-regulation may be a promising therapeutic strategy for the treatment of cancer and that might be associated with PA development, as well.

We began our study by defining *NDRG2* promoter methylation status in PA samples, including all the clinically functioning and hormonally inactive types. We have determined 22.69% (27/119) *NDRG2* methylation frequency. These results indicate that *NDRG2* gene has low methylation status in PAs. A number of studies using various techniques have shown that epigenetic silencing of the *NDRG2* promoter has been found in the majority of primary tumors, and different cancer cell lines and other tumor tissues such as glioma (46.3 - 62%), primary gastric (54%) and colorectal carcinoma (64.28%) cancers [8–11]. *NDRG2* promoter methylation was observed to be associated with the invasiveness in gastric and colorectal cancer and with the aggressiveness of glioma tumor [8–10]. However, our analysis has hown no significant association between *NDRG2* gene methylation and pituitary adenoma invasiveness. Meanwhile, we have demonstrated that hypersecretion of PRL, IGF-1, GH, ACTH hormones appear with a methylated *NDRG2* gene more often than with an unmethylated gene. Also, in most cases nonfunctioning PAs have an unmethylated *NDRG2* gene. However, the mechanisms related to this are still unknown.

Moreover, previous studies have shown that *NDRG2* mRNA expression is low in numerous types of tumor tissues and cancer cell lines, and is a novel tumor suppressor candidate gene [8, 12–16]. It was observed that *NDRG2* expression loss is significantly correlated with aggressive tumor behaviors such as late tumor-node-metastasis stage, differentiation grade, portal vein thrombi, infiltrative growth pattern, nodal/distant metastasis, as well as shorter patient survival rates in liver cancer [13]. Also, *NDRG2* overexpression can inhibit tumor growth and invasion in vitro in bladder and breast cancer [12, 15]. Meanwhile, our results have showed no correlation between *NDRG2* gene mRNA expression and pituitary adenoma invasiveness. The mechanism of *NDRG2* expression in pituitary adenoma proliferation and invasion has not yet been reported, making it necessary to further elucidate the role of *NDRG2* gene in pathogenesis of PA.

In addition, we have analyzed the associations of *NDRG2* gene mRNA expression with clinical features of PAs. Our study has revealed that in the case of acromegaly, *NDRG2* gene mRNA expression is significantly lower than in other diagnoses of PAs. It is known that acromegaly is an insidious disorder characterized by excess secretion of growth hormone and elevated circulating levels of insulin-like growth factor-I [30], and in our examination, in most cases the hypersecretion of GH and IGF-1 hormones were determined with low *NDRG2* gene expression level as well. Moreover, *NDRG2* gene mRNA expression is significantly higher with diagnoses of prolactinoma than in other diagnoses of PAs, and in most cases the hypersecretion of PRL hormone that causes prolactinoma have been detected with medium *NDRG2* expression level. These results are consistent with previous reports that the expression of *NDRG2* is regulated by many hormones, including adrenal steroids [31], dexamethasone [32], insulin [33], androgen [34] and aldosterone [35]. It was also shown that hormone estrogen can enhance the expression of *NDRG2* and may influence Na+/K + -ATPase activation as well as ion transport in salivary glands, brain, heart, skeletal muscle, and kidney where Na+/K + -ATPases were enriched [36]. Also, in human and in animal models estrogen stimulates PRL secretion in vitro and induces PRL adenomas in vivo [37]. However, more studies for signal pathway are needed to show the mechanism underlying and the significant results we showed in this study.

## Conclusion

This is the first study that has demonstrated the *NDRG2* gene promoter methylation and mRNA expression in patients with diagnoses of pituitary adenoma and analyzed the relationships between *NDRG2* epigenetic changes and the association with PA clinical features including patient age, gender, relapse, hormone groups, invasiveness, diagnoses of prolactinoma, acromegaly and Cushing syndrome. Our data have revealed that in the case of acromegaly, *NDRG2* gene mRNA expression is significantly lower than in other diagnosis of PAs and PA that secretes hormones GH and IGF-1 hormones have low *NDRG2* gene expression level as well. Moreover, *NDRG2* gene expression is significantly higher with diagnoses of prolactinoma than in other diagnosis of PAs. Therefore, there is need for intensive research to confirm our findings and justify the hypothesis that *NDRG2* could be a diagnostic marker for diagnosis of prolactinoma and acromegaly in PAs.

## Abbreviations

ACTH: Adrenocorticotropic hormone
COR: Cortisol
CRC: Colorectal cancer
Ct: Threshold cycle
GH: Growth hormone
IGF-1: Insulin-like growth factor 1
MR: Magnetic resonance
MS-PCR: Methylation-specific polymerase chain reaction
*NDRG2*: N-myc downstream-regulated gene 2
PA: Pituitary adenoma
PRL: Prolactin
qRT-PCR: Quantitative real-time polymerase chain reaction
RHB: Human brain reference RNA.
